# *n*-Hexane intoxication in a Chinese medicine pharmaceutical plant: a case report

**DOI:** 10.1186/s13256-017-1280-9

**Published:** 2017-04-28

**Authors:** Jo-Hui Pan, Chiung-Yu Peng, Chung-Ting Lo, Chia-Yen Dai, Chao-Ling Wang, Hung-Yi Chuang

**Affiliations:** 1 Department of Occupational & Environmental Medicine, Kaohsiung Medical University Hospital, Kaohsiung, Taiwan; 2 Department of Public Health, College of Health Sciences, Kaohsiung Medical University, Kaohsiung, Taiwan; 3 Department of Internal Medicine, School of Medicine, Kaohsiung Medical University, Kaohsiung, Taiwan

**Keywords:** *n*-hexane, Neuropathy, Chinese medicine pharmaceutical plant, Occupational exposure, Solvent

## Abstract

**Background:**

*n*-Hexane is a well-known neurotoxicant. Polyneuropathy due to occupational *n*-hexane exposure has been reported worldwide, however, our case is the first report in the Chinese herb industry.

**Case presentation:**

A 25-year-old Asian man experienced progressive weakness and numbness in his hands and feet after working as an operator in a Chinese medicine pharmaceutical plant for the manufacture of Chinese herbal pain relief patches for 10 months. Electrophysiological studies indicated a reduction in nerve conduction velocity, prolongation of distal latencies, mildly positive sharp waves, and reduced recruitment with polyphasic potentials, particularly at distal sites. Demyelination with axonal degeneration caused by occupational *n*-hexane exposure was strongly suspected. Through investigation of our patient’s workplace, the ambient *n*-hexane concentration in air was found to considerably exceed the permissible exposure limit/time-weighted average for *n*-hexane in Taiwan. His symptoms were gradually relieved after 4 months of cessation of exposure to *n*-hexane. He was then confirmed as a case of occupational *n*-hexane intoxication. Further effective control measures should be implemented as soon as possible to prevent exposure of workers to *n*-hexane.

**Conclusions:**

Despite a typical clinical presentation, his exposure at workplace was appropriately investigated. Chemical exposure in Chinese medicine pharmaceutical plants could be an emerging issue that may affect workers’ health. The lack of knowledge and management of solvents could endanger the health of workers. This case has profound educational implications for occupational health and is worthy of further follow-up for improving hazards control.

## Background

*n*-Hexane, with a molecular formula of C_6_H_14_, is a colorless liquid with a disagreeable odor. It is miscible with lipophilic organic solvents and is extremely volatile; therefore, *n*-hexane is widely applied in the production of adhesives, inks, and lacquers, and coating and cleaning agents. It is also used to extract vegetable oils for human consumption and as a substitute for benzene in solvent applications [1]. In addition to concerns regarding its inflammable and explosive nature, *n*-hexane is notorious for its neurotoxicity.

In the 1960s, several cases of *n*-hexane-induced polyneuropathy were reported in poorly ventilated polyethylene laminating plants and a pharmaceutical plant in Japan [2]. Subsequently, this neurotoxic disease was found to be associated worldwide with occupations such as furniture making [3], printing [4], shoe or bag manufacturing [5–7], electronic device manufacturing [8], or other types of work where *n*-hexane-containing adhesive agents were applied [9–12]. Outbreaks were also reported in Taiwan in workers engaged in press proofing [13], ball manufacturing [14], and printing [15].

Due to its known neurotoxicity, preventive measures have been implemented in most industrialized countries. The use of *n*-hexane is declining, and it is largely being replaced with other less toxic solvents [16]. However, occupational *n*-hexane intoxication continues to occur in Taiwan occasionally. The presented case is the first case in the Chinese herb industry in Taiwan. Chemical exposure in Chinese medicine pharmaceutical plants could be an emerging issue that may endanger the health of workers.

## Case presentation

A 25-year-old Asian man was referred to us for further investigation of the relationship between his disease and occupational exposure 5 months after receiving a diagnosis of polyneuropathy at another hospital. Information about his clinical course was mainly obtained from his self-report and medical records.

### Clinical course

A 25-year-old Asian man presented with complaints of progressive weakness and numbness in his hands and feet for the past 10 months. He initially experienced numbness of his right hand. The numbness and weakness spread to all four limbs in 8 months. In the subsequent 2 months, he faced difficulties in using chopsticks, ascending stairs, jumping, and running because of weakness. He denied intake of alcohol or any prescription drugs. On examination, he was conscious and oriented to time, place, and person. He had sensory impairments in a stocking-and-glove distribution, impaired distal muscle power in all four limbs, and absent deep tendon reflex on biceps, ankles, and knees. His cranial nerves were intact. The results of all routine hematological (complete blood cell), serological (anti-nuclear antibody), and biochemical (blood urea nitrogen, creatine, sodium, potassium, glucose, glycated hemoglobin, vitamin B12, and folic acid) examinations were all within normal limits. Results of the tests determining lead, arsenic, and mercury levels in blood samples and cadmium level in urine sample were negative. Electromyography revealed a mildly positive sharp waves in the anterior tibialis and extensor digitorum brevis, and reduced recruitment with polyphasic potentials in the anterior tibialis, extensor digitorum brevis, rectus femoris, and first dorsal interosseous. Nerve conduction velocity studies indicated slowing of motor nerve conduction velocity, and prolongation of distal motor and sensory latencies. Brief data from the medical records at the other hospital are summarized in Tables 1 and 2. Both auditory-evoked potential and visual-evoked potential studies remained normal.

**Table 1:** Electromyography data

Side	Muscle tested	Insertional activity	Spontaneous activity		Motor unit			Recruitment	Interference
			Fibs	Psw	Amp	Dur	Poly		
Right	Anterior tibialis	-	+/-	+/-	-	-	3+	↓	-
Right	Extensor digitorum brevis	-	2+	2+	-	-	3+	↓	-
Right	Rectus femoris	-	-	-	-	-	2+	↓	-
Right	First dorsal interosseous	-	-	-	-	-	3+	↓	-
Right	Biceps (long head)	-	-	-	-	-	0	-	-

- negative finding, *Amp* amplitude, *Dur* duration, *Fibs* fibrillation potentials, *Poly* polyphasic potentials, *Psw* positive sharp waves, ↓ reduced

**Table 2:** Nerve conduction velocity data

Motor nerve conduction studies					Sensory nerve conduction study				
Nerve	Segment	Latency (ms)	Amplitude (μV)	Velocity (m/s)	Nerve	Site	Latency (ms)	Amplitude (μV)	Velocity (m/s)
Median (left/right)	W	6.7/6.6	5.4/4.7		Median (left/right)	W	4.4/4.8	33.7/25.1	NA
	W-E	11.2/11.1	4.2/4.4	42/44		P	2.0/2.1	60.2/55.1	NA
	F-wave	31.16/32.67			Ulnar (left/right)	W	3.9/3.7	22.7/18.5	NA
Ulnar (left/right)	W	4.7/4.8	6.6/4.1						
	W-E	8.5/8.5	6.0/3.4	42/43	Radial (left/right)	W	3.3/3.4	20.1/15.7	NA
	BE-AE	10.7/10.9	6.1/3.3	45/42					
	F-wave	30.54/30.66			Sural (left/right)	C	4.5/4.5	6.1/7.2	NA
Radial (left/right)	BE	2.3/2.3	5.8/5.4						
	BE-AE	4.8/4.8	6.0/4.7	40/40	H reflex study				
Peroneal (left/right)	A	7.8/7.7	1.5/2.1		Nerve			Latency (ms)	
	A-BF	18.3/18.2	1.0/0.4	28/29	Tibial (left/right)			NP/NP	
	F-wave	53.9/NP							
Tibial (left/right)	A	5.6/5.9	5.7/6.1						
	A-K	18.5/18.1	1.9/2.3	31/32					
	F-wave	64.82/64.35							

*A* ankle, *AE* above elbow, *BE* below elbow, *BF* below fibular head, *C* calf, *E* elbow, *K* knee, *NA* not available, *NP* no potential evoked, *P* palm, *W* wrist

**Fig. 1:**
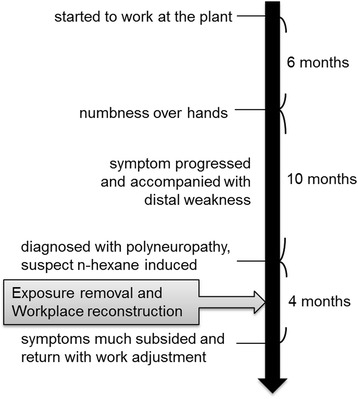
Timeline of the clinical course of the case. The patient’s symptoms began with numbness of his hands 6 months after starting a job. His symptom progressed and was accompanied with distal weakness in the following 10 months. After removal of occupational exposure to *n*-hexane for 4 months, his symptoms subsided and he returned to work in another section of the plant that had no *n*-hexane exposure

Detailed history taking revealed that our patient was exposed to *n*-hexane in his workplace. Demyelination with axonal degeneration caused by occupational *n*-hexane exposure was strongly suspected. He was treated symptomatically with neurotropic vitamins. After removal from exposure for 4 months, his symptoms improved gradually, but he still faced some difficulty in running. Finally, he returned to the plant and shifted to another job without *n*-hexane exposure. The timeline of this case is depicted in Fig. 1.

### Workplace investigation

**Fig. 2:**
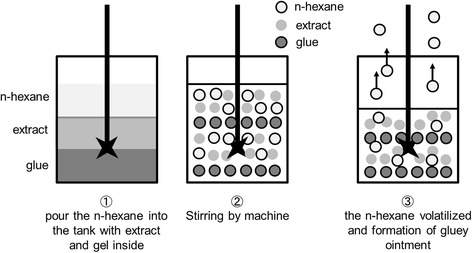
The role of *n*-hexane in the mixing process. ➀ Pour the *n*-hexane into the tank with extract and gel inside. ➁ Stirring by machine. ➂ The *n*-hexane volatilized and formation of gluey ointment

Our patient was an operator in a Chinese medicine pharmaceutical plant for the manufacture of Chinese herbal pain relief patches. For the past 18 months, he had been working in the mixing room to prepare the herbal ointment of the patches. He used to work with bare hands for fear that the gloves might drop into the stirring barrel tank. Because the mixing room was extremely hot and stuffy, he seldom used the respiratory protective mask at work. His first task was to pour *n*-hexane-containing solvents into a barrel tank containing the Chinese herb extract and gel. *n*-Hexane is a solvent and an adhesive used for mixing herbal extract to form a gluey ointment. During the mixing process, *n*-hexane would vaporize and only the gluey ointment would remain (see Fig. 2). His next task was to scoop out the mixture and to separate it into trays. The trays filled with the mixture were then subjected to the next process. Before the next cycle, he had to clean the mixer tank by using a rag dipped in a *n*-hexane-containing solvent. Furthermore, he used the same solvent, instead of water and soap, to clean his hands. The *n*-hexane-containing solvent was stored in buckets without any label or a lid. The daily consumption of the solvent was approximately 180 liters.

He worked in such conditions for at least 6 hours a day, had a 30-minute lunchbreak in between, and had only 4 days off per month. Among the 105 workers in this plant in total, three people, including this patient, were involved in the mixing process. The other operators had been working for 5 to 10 years, none of them exhibited symptoms of neuropathy; however, they had nonspecific complaints like headache, dizziness, and fatigue. Both of the senior workers claimed that they had worn masks and gloves while handling organic compounds all the time at work. The supervisors stated that they only knew that the chemicals used could be toxic. The workers were not allowed to eat or smoke in the workplace. The employer did not provide warning labels, display safety data sheets, ambient air sampling, biological monitoring, or provide the necessary safety and health education and training for workers regarding this toxic chemical. Moreover, no specific measures had been adopted to reduce the risk of solvent exposure.

**Fig. 3:**
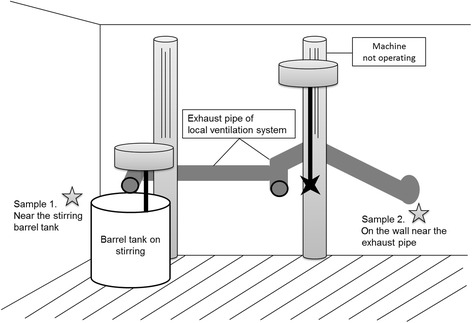
The sampling sites in the workplace. There were four mixers in the mixing room, but only one is working. We placed sample 1 near the stirring barrel tank, sample 2 near the exhaust pipe, and sample 3 on the operator

The mixing room was reconstructed and fitted with an air conditioner and local exhaust ventilating system before our visit. The inlet of the exhaust pipe was near the stirring barrel tank, but there was no hood on it. We took both personal and workplace samples using SKC activated charcoal tubes (SKC catalog number 226-01, 6-mm outside diameter, 100/50-mg sections) under 27 °C and 756 mmHg for 1 hour at the flowrate of 200 mL/minute. The samples were desorbed with carbon disulfide and analyzed through gas chromatography (GC) and mass spectrometry (MS). The sampling sites and results are depicted in Fig. 3 and Table 3.

**Table 3:** The sampling result of *n*-hexane concentration in the air in the mixing room

Sample number	Sampling type	Flow rate (ml/minute)	Sampling time (minutes)	Sampling volume (m^3^)		Adsorbed mass (mg)	Concentration	
				During sampling	NTP		(mg/ m^3^)	(ppm)
1	Workplace^a^	100.0	59	0.005900	0.005836	36.79	6304	1789
2	Workplace^b^	100.6	49	0.004929	0.004876	7.83	1607	456
3	Personal	98.4	59	0.005806	0.005742	2.42	422	120

*NTP* normal temperature and pressure. ^a^This sample placed near the stirring barrel tank had breakthrough. ^b^This sample was placed on the wall near the exhaust pipe

The ambient hexane levels were as high as 1789 ppm and 456 ppm in the mixing room, and 120 ppm for personal concentration. Although the permissible exposure limit (PEL) of Occupational Safety and Health Administration (OSHA) and the recommended exposure level (REL) of the National Institute for Occupational Safety and Health (NIOSH) for *n*-hexane are 500 ppm (1800 mg/m^3^) [17], these three samples considerably exceeded the permissible level in Taiwan, 50 ppm (180 mg/m^3^), which is the same level as the threshold limit value (TLV)-time-weighted average (TWA) of the American Conference of Governmental Industrial Hygienists (ACGIH) [18]. The European Commission recommends that the occupational exposure limit of *n*-hexane is 20 ppm (72 mg/m^3^) [19]. Our findings suggested that workers might be overexposed to hexane even after reconstruction; however, the level of exposure was considerably higher before the reconstruction than after it. Further effective control measures needed to be implemented as soon as possible. The employer promised to make efforts to reduce workers’ exposure to hexane in the future.

## Discussion

Considering the clinical manifestations, high levels of *n*-hexane in the workplace, appropriate temporality of the relationship between exposure and disease, and exclusion of other causes, our patient’s polyneuropathy was closely associated with occupational exposure to *n*-hexane.

Although polyneuropathy due to occupational *n*-hexane exposure has been reported worldwide, this is the first report in the Chinese herb industry in Taiwan. Herbal medicines have been commonly used for health promotion and the treatment of diseases in Asia and Africa for centuries. Preparation of herbal formulations has become a special industry in modern times with the advent of scientific and industrialized manufacturing processes. Many studies are available on the safety of herbal medicines for consumers; however, occupational chemical exposure during preparation also presents a crucial topic for investigation.

The neurotoxicity of *n*-hexane may occur in both the peripheral nervous system and central nervous system (CNS). Peripheral neuropathy is characterized by symmetrical progressive distal sensory and motor impairment. Mostly, the initial symptoms are numbness and a burning sensation in the toes and fingers, followed by distal limb muscle weakness [1]. Axonal degeneration and focal demyelination can be detected using electrophysiological studies with findings such as reduction of nerve conduction velocity, focal conduction block, prolongation of distal latencies, and amplitude reduction of compound muscle action potentials, particularly in the lower extremities [7, 8, 20, 21]. Spontaneous activities, such as fast firing and high-amplitude polyphasic motor unit potentials, are common [1]. CNS conduction abnormalities can be detected in evoked potential studies, or assessed by calculating transcranial magnetic stimulation and spinal nerve root stimulation [21, 22]. Some patients revealed signs of CNS dysfunction such as spasticity of the lower limbs and increasing deep tendon reflexes [23]. Our patient had typical clinical presentations and electrophysiological abnormalities, but without evidence of CNS involvement.

A potential neurotoxic metabolite of *n*-hexane is 2,5-hexanedione (2,5-HD), which acts as a biomarker associated with *n*-hexane exposure [24]. Good correlation was found between the TWA *n*-hexane air concentration and end-of-shift 2,5-HD level in the urine of the patient [25]. The recommended biological exposure index (BEI) value of the ACGIH for the free form of 2,5-HD in urine is 0.4 mg/L, corresponding to a *n*-hexane exposure less than TWA 50 ppm [18]. The 2,5-HD was not a convenient biomarker because the pretreatment of the urine sample is quite complex, and the measurement of 2,5-HD requires advanced instruments, such as GC-MS and adept laboratory workers [25]. The aminoderived pyrroles and thiol-pyrrole conjugates in urine are potential alternative biomarkers of *n*-hexane exposure in the current study [26, 27].

The prognosis of *n*-hexane-induced neuropathy is believed to be good and tends to be biphasic with “coasting” of 2 to 3 months, followed by a slow recovery for approximately 1 to 2 years after the cessation of exposure to *n*-hexane [1]. In the electrophysiological follow-up of 25 patients with chronic peripheral neuropathy induced by occupational *n*-hexane exposure, the recovery of motor nerves was superior to that of the sensory nerves, and upper limbs recovered faster than the lower limbs [8]. Cessation of exposure is the only way to treat *n*-hexane-induced polyneuropathy. Although we had no further electrophysiological follow-up of our case, his symptoms had recovered gradually after cessation of exposure. Detailed history taking is essential not only for early recognition of occupational intoxication, but also to prevent workers from developing further adverse effects.

The lack of a lumbar puncture survey and biomarker data are limitations of this case; however, the diagnosis of *n*-hexane-induced peripheral polyneuropathy is not controversial. We have strong evidence of occupational exposure and our patient’s recovery after removal of exposure.

To prevent similar occurrences, the use of toxic chemicals or substances should be well managed. We must consider that no chemical is safe. Risk assessment, risk ranking management procedures, workplace monitoring, chemical handling training, and health education should be included in the management of chemicals or toxic substances. Employers, chemical importers, or suppliers cannot provide any excuse for a lack of knowledge regarding the chemicals used on-site, including their properties and hazards. They should take complete responsibility for following the instruction for hazard control and should protect the health of workers.

Poor ventilation in the workplace has been one of the major factors in almost all cases reported worldwide [25], and a similar situation was also found in our case. Although the room had been reconstructed with a ventilation system, the workplace *n*-hexane levels considerably exceeded the PEL. Industrial control of exposure includes not only the installing of ventilation equipment but also making it work effectively. There are two control alternatives in the case, namely enclosing the mixing tank and venting out hexane vapors and installing a proper hood at the end of the exhaust pipe.

Personal protective equipment is the least line of protection for workers’ health from exposure to various hazards. *n*-Hexane is present as liquid or vapor under normal temperature and pressure; thus, workers were exposed to *n*-hexane in the workplace through inhalation and dermal contact. In our case, workers were provided with a mask and gloves for protection from organic compounds; however, our patient avoided using them. The employer or the supervisor should undertake the responsibility of supervising and educating workers. Workers should be instructed not only to wear tight-fitting masks in the mixing room, but also to replace or repair the respirator if vapor or gas leaks are detected.

## Conclusions

In conclusion, we have obtained compelling evidence of exposure through workplace investigation. Chemical exposure in Chinese medicine pharmaceutical plants could be an emerging issue that may affect workers’ health. Insufficient knowledge and management of *n*-hexane could endanger the health of workers. This case has profound educational implications for occupational health and is worthy of further follow-up for improving hazards control.

## Abbreviations

2,5-HD: 2,5-Hexanedione
ACGIH: American Conference of Governmental Industrial Hygienists
BEI: Biological exposure index
CNS: Central nervous system
GC: Gas chromatography
MS: Mass spectrometry
NIOSH: National Institute for Occupational Safety and Health
OSHA: Occupational Safety and Health Administration
PEL: Permissible exposure limit
REL: Recommended exposure level
TLV: Threshold limit value
TWA: Time-weighted average
